# Stochastic models for objects and images in oncology and virology: application to PI3K-Akt-mTOR signaling and COVID-19 disease

**DOI:** 10.1117/1.JMI.8.S1.S16001

**Published:** 2020-11-26

**Authors:** Harrison H. Barrett, Luca Caucci

**Affiliations:** a University of Arizona, Wyant College of Optical Sciences, Tucson, Arizona, United States; b University of Arizona, Department of Medical Imaging, Arizona, United States

**Keywords:** stochastic models, characteristic functional, mammalian target of rapamycin signaling, coronavirus, lipid rafts

## Abstract

**Purpose:** The goal of this research is to develop innovative methods of acquiring simultaneous multidimensional molecular images of several different physiological random processes (PRPs) that might all be active in a particular disease such as COVID-19.

**Approach:** Our study is part of an ongoing effort at the University of Arizona to derive biologically accurate yet mathematically tractable models of the objects of interest in molecular imaging and of the images they produce. In both cases, the models are fully stochastic, in the sense that they provide ways to estimate any estimable property of the object or image. The mathematical tool we use for images is the characteristic function, which can be calculated if the multivariate probability density function for the image data is known. For objects, which are functions of continuous variables rather than discrete pixels or voxels, the characteristic function becomes infinite dimensional, and we refer to it as the characteristic functional.

**Results:** Several innovative mathematical results are derived, in particular for simultaneous imaging of multiple PRPs. Then the application of these methods to cancers that disrupt the mammalian target of rapamycin signaling pathway and to COVID-19 are discussed qualitatively. One reason for choosing these two problems is that they both involve lipid rafts.

**Conclusions:** We found that it was necessary to employ a new algorithm for energy estimation to do simultaneous single-photon emission computerized tomography imaging of a large number of different tracers. With this caveat, however, we expect to be able to acquire and analyze an unprecedented amount of molecular imaging data for an individual COVID patient.

## 1 Introduction

This paper concerns new methods of image acquisition and analysis that can contribute to clinical patient management and scientific investigations for various diseases. The mathematical underpinnings of the methods have been developed in many papers and illustrated by simulation studies of various molecular imaging modalities, but there is a need for implementations that can be used with real patient data and real clinical objectives.

We have chosen to illustrate these details by considering two quite different disease types: cancers that involve mutations on the mammalian target of rapamycin (mTOR) pathway, and coronovirus infections (specifically COVID-19). The former has the advantage that the biophysics of disease progression is well understood, while the latter will highlight some of the uncertainties in the biophysics and how they can be resolved.

Throughout this paper, it will be assumed that the object being imaged is also the source of the radiation that forms the image. We also assume that the imaging systems are tomographic, meaning that they acquire information about the 3D structure of the object. Thus, we are in the realm of emission computed tomography (ECT), which includes positron emission tomography (PET), single-photon emission computed tomography (SPECT), various forms of optical fluorescent imaging, and the new modalities of charged-particle emission computed tomography (CPET) for alpha and beta particles.^1^ We do not consider computed tomography, magnetic resonance imaging, or indirect optical methods such as fluorescence lifetime imaging.

The main mathematical tools to be utilized are characteristic functionals and characteristic functions, to be defined in the next section; these tools lead to rigorous mathematical and statistical descriptions for physiological objects and their molecular images.

## 2 Statistical Concepts and Notation

### 2.1 Digital Images

A digital image, virtually by definition, is a finite set of numbers; were this not the case, the image could not be stored and processed in a computer or displayed on a monitor. In tomographic imaging, it is common to associate a volume element (voxel) with every point in the digital image. In mathematical terms, the digital image is a vector in an M-dimensional vector space, where M is the number of voxels available to describe the image. Equivalently, the image can be treated as an (M×1) column vector, denoted g. We can define the characteristic function of g as

$$\psi_{g}(\xi )=\langle \exp(-2\pi i\xi^{†}g)\rangle ,$$ (1)

where the angle brackets denote a statistical average, and the superscript † denotes an adjoint (complex-conjugate transpose); thus, $\xi^{†}$ is a 1×M row vector, so $\xi^{†}g$ is a scalar product. It is worth noting that $\psi_{g}(\xi )$ is a scalar, but it depends on the vector ξ, which can be chosen at will.

A simple interpretation of $\psi_{g}(\xi )$ is that it is the M-dimensional Fourier transform of the probability density function of the M-dimensional random vector g.

### 2.2 Objects: Physiological Random Processes

Physiology is the study of life. Thus molecular imaging of living beings should provide information about physiological processes. Moreover, very little about life is predictable. Etymologically, the opposite of predictable is stochastic, or random, so the objects being imaged in living beings are physiological random processes (PRPs). Objects are functions of continuous variables, and images are sets of numbers. Technically, objects are vectors in an infinite-dimensional Hilbert space, and images are vectors in a finite-dimensional Euclidean space.

The simplest form of characteristic functional^2^ of a PRP is [cf. Eq. (1)]

$$\Psi_{f}(\Phi )=\langle \exp[-2\pi i(\Phi ,f)]\rangle ,$$ (2)

where f is a shorthand for a function of three spatial variables, f=f(r), and r=(x,y,z). Likewise, Φ is also a function of three spatial variables, hence, a function in the same vector space as f, and the scalar product is defined as

$$(\Phi ,f)=\int_{V}d^{3}r\Phi^{*}(r)f(r),$$ (3)

where V is the volume of integration, asterisk denotes a complex conjugate, and $d^{3}r=dxdydz$.

### 2.3 From Objects to Images

It is often very easy to compute the characteristic function for an image if the characteristic functional of the object is known. As an example, consider a continuous-to-discrete linear operator H. In the absence of excess detector noise, the imaging equation is g=Hf. If we let ξ=HΦ in Eq. (1), we have

$$\psi_{g}(\xi )=\Psi_{f}(H^{†}\xi ).$$ (4)

Thus, a simple change of variables in the argument converts $\Psi_{f}$ to $\psi_{g}$, thereby converting a characteristic functional to a characteristic function. The operator $H^{†}$ is referred to as back-projection in the tomography literature.

### 2.4 Multiple PRPs

In many situations, it is useful to consider simultaneous ECT imaging of multiple, interacting PRPs. Much of the current research in molecular imaging revolves around the search for imaging biomarkers, defined loosely as a way to make a PRP apparent in a medical image. Intuitively, multiple biomarkers will be better than one, and a group of biomarkers will be more valuable still if there is a convincing theory that ties them together (see, for example, Henscheid et al.^3^).

As an example of a biological system with multiple PRPs where the characteristic functional can be expressed fairly succinctly, consider the basic problem of chemotherapy.^3^^,^^4^ Here, the drug is usually administered intravenously, and it is transported into the tumor vasculature where it encounters a dense and complicated network of capillaries. Many papers in the systems-biology literature attempt to construct models of the capillaries, but the outcomes are seldom realistic, and virtually never do they capture the intricacies of the vasculature for a particular patient.

Minimally, at least three PRPs are needed to describe the drug distribution in the context of chemotherapy: the concentration of drug in the capillaries as a function of space and time; the vascular permeability as a function of position in the tumor, and the process where a drug molecule emerges from a capillary, diffuses to a tumor cell, and binds to it.

If we let $f_{1}$ represent the drug concentration, $f_{2}$ the vascular permeability and $f_{3}$ the drug diffusion in the interstitium, respectively, then the form of the characteristic functional is

$$\Psi_{F}(\Phi )={\left\langle \langle \langle \exp-2\pi i[(\Phi_{1},f_{1})+(\Phi_{2},f_{2})+(\Phi_{3},f_{3})]\right\rangle }_{f_{1}|f_{2},f_{3}}\rangle_{f_{2}|f_{3}}\rangle_{f_{3}},$$ (5)

where F is the concatenation of the three terms and the subscripts on the angle brackets denote conditional averages; for example, ${\left\langle \cdots \right\rangle }_{f_{1}|f_{2},f_{3}}$ denotes an average over $f_{1}$ with $f_{2}$ and $f_{3}$ held constant. It is worth noting that the order of averaging follows the physical flow of the therapy drug. In other words, there is no feedback in Eq. (5). In Sec. 3, we examine a pathway where feedback plays a critical role.

### 2.5 Systems Biology

Systems biology is a popular field of current research that often appears as a submission category in scientific meetings or biology journals. The mathematical models customarily used in systems biology can be (a) sets of coupled ordinary differential equations (ODEs); (b) sets of coupled partial differential equations (PDEs); (c) Boolean operators as in computer science, or (d) combinations of these objects. A recent review article that illustrates some of these choices in the context of the mTOR pathway is by Sulaimanov et al.^5^

If ODEs are used, the variable of interest is almost always time, and we are in the realm of compartmental modeling. The use of both spatial and temporal variables, hence PDEs, is more realistic, but requires vastly more unknown parameters.

One fairly obvious way to study PRPs in a laboratory or clinic is to use monoclonal antibodies designed to bind to particular biomolecules of interest. These antibodies can be used with either fluorescent labels and optical imaging or with radioactive labels and SPECT imaging.

Laboratory studies with real tracers and imaging systems produce far richer data than those available from a systems-biology simulation, especially with multiple PRPs. A single simulation run requires choosing mathematical forms for each PRP as a function or space and/or time, and the whole process must be repeated many times with different assumed forms for each PRP to learn anything about uncertainties in the systems-biology results. From a single real measurement of the characteristic functional, on the other hand, one can compute all means and variances and any other desired statistical properties of the real tracer distributions.

## 3 Signaling Pathways

### 3.1 Structure of an Example Pathway

The pathway of interest here is phosphoinositide-3-kinase (PI3K)/Akt/mTOR, where mTOR stands for mechanistic (or mammalian) TOR.

Rapamycin was isolated in 1972 from bacteria found on Rapa Nui (Easter Island). It was initially developed as an antifungal agent. Rapamycin was soon found to inhibit mTOR, which makes it a potent immunosuppressive and antiproliferative agent. It is used widely to prevent rejection in organ transplantation. Deregulation of its signaling pathways is associated with numerous diseases affecting large populations, including obesity, diabetes, and cancer. The pathway considered here is now regarded as a nearly universal physiological regulator.

A rigorous mathematical model of the PI3K/Akt/mTOR signaling pathway would be invaluable in patient-specific therapy of many cancers. The first step, PI3K, is mediated by a cell-surface receptor called phosphatidylinositol triple kinase. (A kinase is an enzyme that mediates phosphorylation of a biomolecule, a key step in many biological reactions.)

Akt is also known as protein kinase B, and its activity is controlled by a phosphatase-and-tensin homolog called PTEN. (a phosphatase is the opposite of a kinase; it removes a phosphate rather than adding one).

**Fig. 1:**
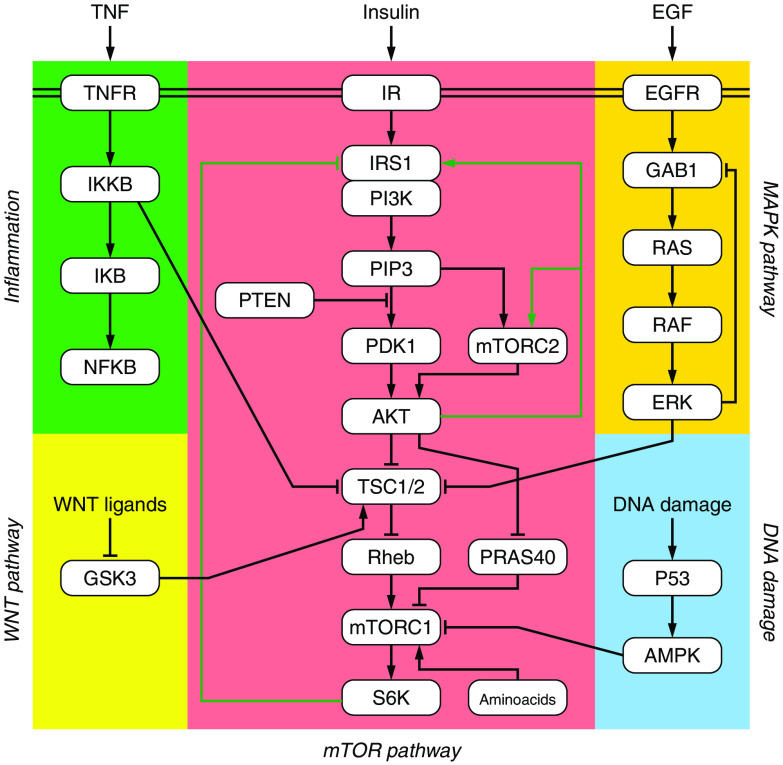
Diagram of the mTOR pathway. The double line at the top of the diagram represents the plasma membrane, and the single green lines are feedback paths. See text for explanation. Reproduced in modified form from Sulaimanov et al.^5^ under the terms of Creative Commons Attribution-NonCommercial 4.0 International Public License (available at Ref. 6). Original creators of the figure and copyright holders: Nurgazy Sulaimanov, Martin Klose, Hauke Busch, and Melanie Boerries.^5^

Further, along in the pathway is a complex of two kinds of mTOR, one of which controls the synthesis of proteins through ribosomal proteins such as S6K. (Ribosomes are molecular machines that translate messenger ribonucleic acid (mRNA) to proteins, and they are themselves partly constructed of proteins.) Other functions of the PI3K/Akt/mTOR pathway include control of apoptosis (programmed cell death) and repair of deoxyribose nucleic acid (DNA) damage. To illustrate the complexity of this system, there can be as many as 10 million ribosomes in a single mammalian cell, and there are approximately a billion cells in a one-gram tumor (Fig. 1).

### 3.2 Lipid Rafts, Pathway Activation, and Phosphorylation Waves

A signaling pathway is said to be activated if it is capable of running continuously without external controls. The PI3K-Akt-mTOR pathway is activated by double phosphorylation of Akt with two different kinases, mTORC2 and PDK1. These actions occur in lipid rafts, which are microdomains in the plasma membrane delineated by regions high in cholesterol and sphingolipids. For more details see Gao et al.^7^

Compartmentalization into microdomains is essential for activation of Akt by PDK1 and mTORC2 (two phosphorylation events). One effect of membrane localization is an increase in concentration of signaling proteins, which leads to a rapid switch-like response.

Moreover, the inhibitory signaling protein PTEN is localized outside the membrane rafts and cannot interfere with the activation. Gao et al.^7^ used genetic engineering to modify the lipids in the raft so that PTEN was no longer excluded; they found that when PTEN could enter the rafts, it “abolished the activity of the whole pathway.” See Gao et al.^7^ again.

Following the double phosphorylation, the activated Akt escapes from the lipid raft with the help of PIP3 and then migrates to ribosomes in the cytoplasm and nucleus. One might expect this migration to be diffusive, but Markevich et al. have shown that the activated Akt molecules actually propagate as phosphorylation waves. These waves are created by the feedback paths shown in the figure above. Markevich et al.^8^ have shown that the positive feedback leads to bistability, with the two states being Akt singly phosphorylated and doubly phosphorylated. The resulting pair of coupled waves then propagate with little attenuation or scattering, greatly enhancing their efficiency in passing their activation on to ribosomes.

It would appear to be straightforward to construct a characteristic functional incorporating all aspects of this pathway. Such a theory would take advantage of the fact that the initiating events, Akt activations, are statistically independent since they take place in isolated lipid rafts, one activation at a time. That means that the whole set of activated Akts is well described as a sample function of a Poisson random process, with a well-known characteristic functional.^9^

## 4 COVID-19 Disease

### 4.1 Introduction

In essence, a virus is a segment of genetic material inside a protein shell. A virus is not a living being, rather a parasite that replicates within a host cell. The particular virus discussed here is an RNA virus called Severe Acute Respiratory Syndrome–CoronaVirus-2 (SARS-CoV-2). The associated disease, called COVID-19, was discovered in December 2019, in Wuhan, China.

By early February 2020, remarkably detailed papers by Chinese scientists had been published (online or in print) in world-renowned scientific journals (*Nature*, *The Lancet*, and NEJM),^10^^–^^12^ barely six weeks after the first cases in Wuhan. It was apparent that the new disease was closely related to SARS and Middle East respiratory symdrome (MERS), hence also a corona virus. By this same time frame, Chinese scientists had determined and published the full genome of the virus, and the World Health Organization (WHO) had declared a pandemic.

### 4.2 Mechanisms of Viral Infection

The early Chinese papers established that the probable entry routes by which a novel coronavirus could infect a cell were essentially the same as for SARS and MERS. In a 2012 review of SARS and MERS, Belouzard et al.^13^ focused on the role of the viral spike protein (S) in mediating cell entry. The process starts with conformational changes of the S protein triggered by receptor binding, pH changes, or other stimuli.

For pulmonary infections, the relevant receptors are the angiotensin converting enzyme 2 (ACE2) and the angiotensin receptor (AR). Each of these is associated with a common class of drugs for hypertension: ACE inhibitors (ACEI) and AR blockers (ARB). Another molecule that is important to the entry process is transmembrane protease serine 2 or TMPRSS2.

As an aside, some early investigators suggested using ACEIs and ARBs in conjunction with other COVID-19 therapy, but there was always some concern about interfering with normal respiratory function. A few recent clinical trials have shown, however, that there may be some benefit in continuing with hypertension drugs during any therapy on hypertensives.

Returning to mechanisms of coronavirus infection, it is not completely clear what happens after a virus enters a cell at an ACE2 or AR site. Wang et al.^14^ show fairly convincingly that entry is facilitated by high levels of cholesterol, which they presume to be stored in lipid rafts in the plasma membrane. These lipid rafts, however, do not have the same physical structure as the ones we discussed in Sec. 3.2 in the context of the mTOR pathway. The latter is anchored by transmembrane receptors, and they are delineated by high levels of cholesterol and sphingolipids that serve the important function of keeping the inhibitory protein PTEN out of the raft. The rafts of interest for COVID-19 are not so sharply delineated; they can migrate laterally on the membrane and merge to form larger rafts. On the other hand, they can serve as cholesterol repositories, thereby accounting for the increased susceptivity to COVID-19 for older patients.

### 4.3 Inflammation, Cytokine Storm

There is considerable discussion online about how to define disease mortality, but an intuitive practical definition is number of deaths divided by the number of confirmed cases; by this token the mortality of COVID-19 in the US is about 5%. This number is substantially higher for elderly patients or those with comorbidities, and it is much less for children.

For all ages, it appears that a large fraction of the deaths is the result of cytokine storms, or colloquially, the immune system run amok.

A cytokine is a signaling molecule that initiates the movement of other molecules. The cytokines known to be relevant to COVID-19 include several interleukins, vascular endothelial growth factor A, tumor necrosis factor, and monocyte chemoattractant protein 1 (MCP1). For many more details, see Nile et al.^15^

Of these cytokines, perhaps the easiest to understand is MCP1, also known as C-C motif ligand 2. A monocyte is a leukocyte (white blood cell), but a primitive one very early in its development. An inflamed tissue can send out MCP1 and receive a cloud of monocytes in return; the monocytes then differentiate into tissue-resident macrophages, which can engulf inflamed cells. One way this process can stop is by further differentiation to foam cells, but little is known about the efficiency of this step. If the process does not stop, a cytokine storm ensues.

Within the context of this paper, molecular imaging might be a good tool for early detection of cytokine storms.

### 4.4 Vaccines and Therapeutics

Anything approaching a comprehensive discussion of the worldwide search for COVID-19 vaccines or therapeutics is far beyond the scope of this paper, but a readable and informative survey is given by Timmer.^16^ The Timmer paper might profitably be read following the present paper.

### 4.5 Role of Characteristic Functionals

So far we have treated characteristic functionals mainly as tools for estimating stochastic properties of PRPs, but they can also play a role in hypothesis testing. Suppose, for example, we use K radioactive tracers with different energies to study K PRPs. The vector Φ then signifies K separate functions of three spatial dimensions, one for each energy. If K is large, we will need an imaging gamma-ray detector with excellent energy resolution to acquire K separate SPECT images. It would not suffice to have a narrow photopeak; we also have to suppress spurious peaks arising from escape of fluorescent X rays or Compton-scattered photons.

A general method for suppressing these spurious peaks is based on the maximum-likelihood expectation-maximization algorithm as applied to energy spectra.^17^ With this tool and any reasonably good SPECT system, we can use the vector Φ to scan the reconstructed images for missing peaks or unexpected peaks that could represent potential therapeutic targets for that specific patient. This procedure can be viewed as testing the dual hypothesis that all relevant proteins have been included in the mathematical model and none have been missed.

## 5 Example of Application of Characteristic Functionals to COVID-19

There are many possible applications in COVID-19 virology to which we could apply ECT and characteristic functionals. For each, we would have to define an important biological or clinical question; choose a relevant ECT imaging modality and associated tracers, and design a data-acquisition strategy. The example provided in this section was inspired by the recent discovery of a new mutated strain of COVID-19 that is 5 to 10 times more infectious than the original strain from Wuhan, yet paradoxically no more lethal.

In a comprehensive worldwide epidemiological study, Korber et al.^18^ established the nature of this mutation, which turns out to be a change in a single amino acid at one particular location in the genome of the spike protein. According to Korber et al.,^18^ when the SARS-COV-2 virus emerged in Europe in January 2020, almost 100% of clinical samples in the study coded for aspartic acid (symbol D) in position 614 in the spike protein. By May 2020, the virus had mutated, at least in Europe, so that the same location now coded for glycine (symbol G) in almost 100% of cases. This transition, denoted Spike D614G, results in a 5-10-fold increase in infectivity.

**Fig. 2:**
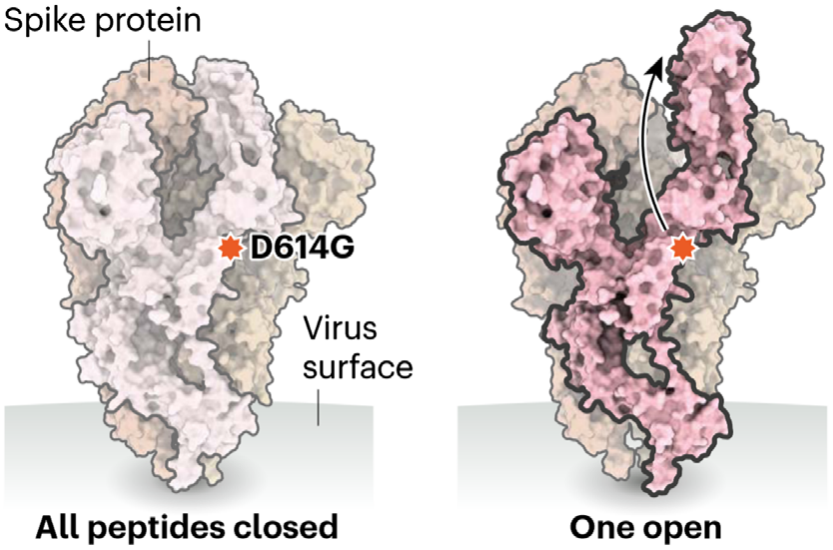
Spike proteins on COVID-19 bind to receptors on human cells, helping the virus to enter. A spike protein is made up of three smaller peptides in “open” or “closed” orientations; when more are open, it is easier for the protein to bind. The D614G mutation seems to relax connections between peptides. Adapted from Ref. 19 with permission.

To see why this seemingly minor genetic modification causes such a major change in viral function, we note that glycine is the only achiral amino acid, which in simple terms means that it can be superimposed on its mirror image by rotations and translations. All other amino acids have complex sidechains that prohibit this superposition; in glycine, the sidechain is just a hydrogen atom. Glycine does not exhibit optical activity and it is neither left-handed nor right-handed. In proteins and other large biomolecules, glycine permits sharp turns in the folded chains (Fig. 2).

Though the D614G mutation described above accounts for the increased infectivity, it does not explain the apparent absence of increased clinical lethality. For this purpose, we need mathematical models of viral cell entry akin to those used by Henscheid et al.^3^ for oncology. Several models of infection and cell entry have appeared in the literature; each is characterized by a small number of proteins. These proteins can be made visible to a fluorescence imaging system by labeling them with fluorescent peptides, with different fluorescence spectra for different proteins.

**Fig. 3:**
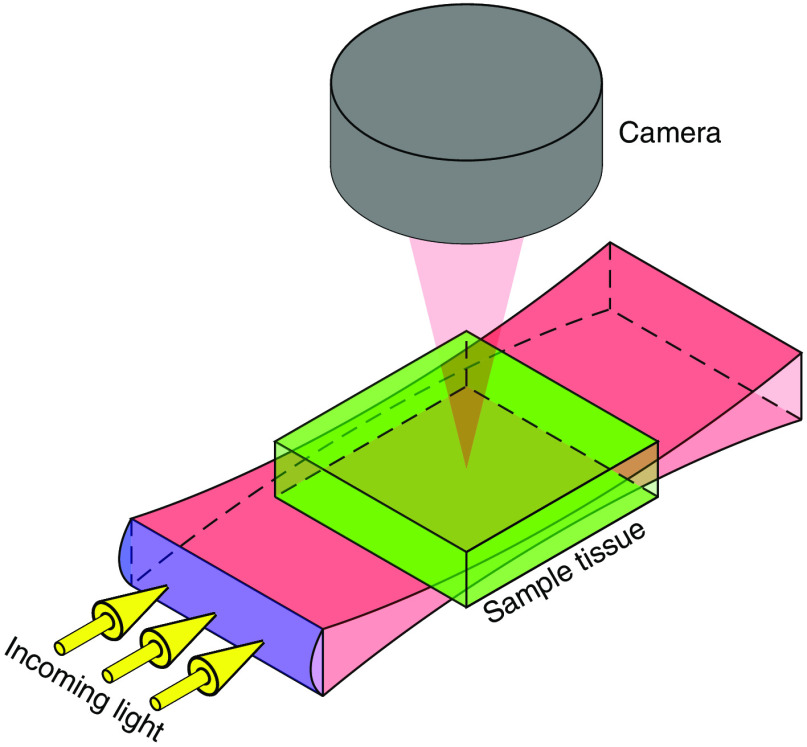
Generic set up of light-sheet fluorescence microscopy. A thin plane of light (shown in red) is created with a cylindrical lens. The plane of light is used to optically section a transparent sample tissue (shown in green). Imaging is done perpendicularly to the direction of illumination.

One very attractive way to image the fluorescent signals is to use a scanning light-sheet (SLS) imaging system like that depicted schematically in Fig. 3. High-end commercial SLS systems have subcellular spatial resolution (≈400  nm), a field of view of about one cubic centimeter, and excellent sensitivity to the fluorescent radiation. One drawback to SLS is that tissue scatters and absorbs optical radiation, but there are many clearing agents available to render tissue transparent.

In the standard operating mode of an SLS imaging system, the various proteins can be depicted in a pseudocolor image, where different colors correspond to different peptides, hence different proteins. The images are remarkable, but no information is obtained concerning the spatial and temporal statistics of the corresponding PRPs. In particular, there is no information about how different PRPs interact with each other, which is key to development of accurate models of system involving several PRPs.

On the other hand, however, SLS imaging can provide a much richer data set than just snapshots. For one thing, it essentially avoids the seemingly insurmountable problem of getting a large number of accurately labeled cases for clinical studies. The logical limit of this conundrum is the challenge of doing trials with a single patient, that is, N=1.

The property of SLS systems that makes all of this possible is their space-bandwidth product, loosely defined as the number of resolvable voxels in the whole data set. With the specifications given above for high-end commercial SLS systems, this number turns out to be about $10^{13}$. Of course, one cannot store data blocks this large, but it is possible to extract essential features from the data on the fly and store them efficiently. For example, we can estimate the centroids of virus particles and use them and associated peptide signals to determine how far along the virus is in the process of binding to a host cell. These features can be used to calculate characteristic functionals [see Eq. (5)].

## 6 Summary and Future Work

All disease processes are enormously complicated, involving many physiological processes, and both patient-to-patient and cell-to-cell variability. We have argued in this paper that characteristic functionals are a systematic tool for understanding interactions among PRPs, even those involving external therapeutic agents. Moreover, PRPs can virtually always be observed directly with *in vivo* molecular imaging. These observations can then be connected to models describing the interactions among random processes, thus potentially verifying the theories or leading to refinements of them. Detailed validation is needed, but this formalism holds great promise as a tool for biomedical research and clinical medicine.

An immediate challenge in this field is to develop efficient methods for estimating patient-specific and disease-specific characteristic functionals for multiple PRPs. An important extension of this study would be to set confidence intervals on estimated variances or other uncertainty measures, taking into account both measurement errors and model inaccuracy. The ultimate performance metric could be a therapy operating characteristic curve as introduced in a previous paper in this paper.

For more details on characteristic functionals, see *Foundations of Image Science*^9^ or the recent review by Clarkson and Barrett.^20^ An extensive treatment of PRPs and characteristic functionals in precision cancer therapy is given by Henscheid et al.^3^
