# Publisher’s Note: Stochastic models for objects and images in oncology and virology: application to PI3K-Akt-mTOR signaling and COVID-19 disease

**DOI:** 10.1117/1.JMI.8.S1.019801

**Published:** 2020-12-17

**Authors:** Harrison H. Barrett, Luca Caucci

**Affiliations:** a University of Arizona, Wyant College of Optical Sciences, Tucson, Arizona, United States; b University of Arizona, Department of Medical Imaging, Arizona, United States

## Abstract

The article was republished with a corrected digital object identifier (DOI).

This article [*J. Med. Imag.* **8**(S1), S16001 (2020) doi: 10.1117/1.JMI.8.S1.S16001 was originally published in Vol. 8, No. S1 of the *Journal of Medical Imaging* (JMI) on 1 December 2020 with an incorrect digital object identifier (DOI) of 10.1117/1.JMI.7.6.S16001. The DOI was corrected and registered as 10.1117/1.JMI.8.S1.S16001 on 3 December 2020.

The typical structure of the DOI for JMI uses the first seven digits to identify the publisher, and the final 8 digits to indicate the volume number, issue, and citation identifier (CID). The corrected DOI indicates that the article was published in Volume 8, Special Issue 1.

